# Z-Scheme Heterojunction of SnS_2_/Bi_2_WO_6_ for Photoreduction of CO_2_ to 100% Alcohol Products by Promoting the Separation of Photogenerated Charges

**DOI:** 10.3390/nano12122030

**Published:** 2022-06-13

**Authors:** Yong Xu, Juanjuan Yu, Jianfei Long, Lingxiao Tu, Weili Dai, Lixia Yang

**Affiliations:** Key Laboratory of Jiangxi Province for Persistent Pollutants Control and Resources Recycle, School of Environmental and Chemical Engineering, Nanchang Hangkong University, Nanchang 330063, China; xu_yong001@163.com (Y.X.); qddxhhh_1@sina.com (J.Y.); 15007047545@139.com (J.L.); 2002085700109@stu.nchu.edu.cn (L.T.); zgay20080808@126.com (L.Y.)

**Keywords:** Bi_2_WO_6_, SnS_2_, photocatalytic CO_2_ reduction, charge separation, liquid phase products

## Abstract

Using sunlight to convert CO_2_ into solar fuel is an ideal solution to both global warming and the energy crisis. The construction of direct Z-scheme heterojunctions is an effective method to overcome the shortcomings of single-component or conventional heterogeneous photocatalysts for photocatalytic CO_2_ (carbon dioxide) reduction. In this work, a composite photocatalyst of narrow-gap SnS_2_ and stable oxide Bi_2_WO_6_ were prepared by a simple hydrothermal method. The combination of Bi_2_WO_6_ and SnS_2_ narrows the bandgap, thereby broadening the absorption edge and increasing the absorption intensity of visible light. Photoluminescence, transient photocurrent, and electrochemical impedance showed that the coupling of SnS_2_ and Bi_2_WO_6_ enhanced the efficiency of photogenerated charge separation. The experimental results show that the electron transfer in the Z-scheme heterojunction of SnS_2_/Bi_2_WO_6_ enables the CO_2_ reduction reactions to take place. The photocatalytic reduction of CO_2_ is carried out in pure water phase without electron donor, and the products are only methanol and ethanol. By constructing a Z-scheme heterojunction, the photocatalytic activity of the SnS_2_/Bi_2_WO_6_ composite was improved to 3.3 times that of pure SnS_2_.

## 1. Introduction

The fossil fuels dilemma has become a serious issue that must be confronted and tried to solve, especially for the demand of industrial development in today’s society [1,2,3]. Inspired by natural photosynthesis, the conversion of CO_2_ into renewable energy driven by light is widely claimed to be the most promising and environmentally friendly way to deal with the global greenhouse effect [4,5,6]. Although a great deal of effort has been made to transform CO_2_ into valuable chemicals, it is rarely reported that the reduction products are alcohol products with high selectivity [5,7]. More importantly, C2 hydrocarbons are hard to form because it is difficult for C-C coupling reaction to occur and more electrons and protons are needed to participate in the reaction. Another important issue is that semiconductor catalysts suffer from the sluggish charge separation efficiency ascribing to the fast recombination of photogenerated electrons and holes during the photoreaction process [8,9,10].

In order to improve the efficiency of charge separation as well as the durability of photocatalysts, a great many measures have been taken including adding cocatalysts, manufacturing defects or doping elements in the bulk materials, forming heterojunction, and so on [11,12,13,14]. The construction of heterojunction can not only improve the light absorption capacity but also enhance the separation efficiency of photogenerated charge. For example, Guo et al. constructed a Z-scheme NiTiO_3_/g-C_3_N_4_ (NT/GCN) photocatalyst by a facile calcination method. In the absence of any sacrificial agent and cocatalyst, the optimized NT/GCN40 can obtain the highest CH_3_OH yield (13.74 μmol∙g^−1^∙h^−1^), which is almost 3.29 times that of g-C_3_N_4_ [15]. Wang et al. designed and fabricated 0D/2D direct Z-scheme heterojunction involving carbonized polymer dots and Bi_4_O_5_Br_2_ nanosheets (CPDs/Bi_4_O_5_Br_2_), which effectively facilitate migration and separation efficiency of photogenerated carriers and retain more negative electron reduction potential of CPDs and more positive hole oxidation potential of Bi_4_O_5_Br_2_. The 8 wt% CPDs/Bi_4_O_5_Br_2_ exhibits the maximal CO production of 132.42 μmol h^−1^g^−1^ under Xe lamp irradiation, 5.43-fold higher than that of Bi_4_O_5_Br_2_ nanosheets [16].

SnS_2_, as a narrow bandgap semiconductor, is nontoxic and has a bandgap value of 2.2–2.4 eV, which can absorb visible light efficiently. It has a CdI_2_-type layered structure, and the force between the layers is the weak van der Waals force. SnS_2_ has good chemical thermal stability and oxidation resistance in acidic and neutral solutions, so it has been favored by researchers as a new type of visible light response photocatalyst in recent years [17]. Wu et al. successfully fabricated the SnS-SnS_2_ Z-scheme heterostructure with nanosheet framework by post-annealing in a specified atmosphere to partially convert the SnS_2_ matrix into SnS. The converted SnS possesses CO_2_ adsorption sites with significantly reduced activation energy, which can be used to drive the rate-determining step for efficient CO_2_ conversion. The final Z-scheme SnS-SnS_2_ heterostructure enhances the photocatalytic activity of CO_2_ conversion to C2 and C3 hydrocarbons [18]. Wu et al. constructed a novel direct Z-scheme g-C_3_N_4_/SnS_2_ heterojunction by in situ deposition of SnS_2_ quantum dots onto the g-C_3_N_4_ surface by a simple one-step hydrothermal method. The transfer of electrons from g-C_3_N_4_ to SnS_2_ results in the formation of an intra-interface electric field (IEF) between the two semiconductors. Compared with g-C_3_N_4_ and SnS_2_ alone, the g-C_3_N_4_/SnS_2_ hybrid showed better performance of photocatalytic CO_2_ reduction [19]. Bi_2_WO_6_ is a semiconductor multi-element oxide with the bandgap of 2.75 eV, which has a certain response under visible light. Bi_2_WO_6_ is a direct bandgap semiconductor, the photogenerated electrons and holes generated by illumination have a high probability of direct recombination. So, the photon quantum efficiency is low. In addition, the Bi_2_WO_6_ material also has a small specific surface area, and the photogenerated charges are easily recombined in the bulk phase, which limits its practical application. According to the energy band matching theory, the energy band positions of SnS_2_ and Bi_2_WO_6_ match each other, and the combination of SnS_2_ and Bi_2_WO_6_ can effectively reduce the recombination of photogenerated electron-hole pairs, thereby achieving the purpose of improving the photocatalytic efficiency. Here, we constructed stable Bi_2_WO_6_ oxide semiconductors and SnS_2_ narrow-bandgap semiconductors as Z-scheme heterojunctions to improve the separation efficiency of photogenerated electrons and holes, thereby enhancing the photocatalytic activity. We also expanded the application of SnS_2_/Bi_2_WO_6_ in photocatalytic reduction of CO_2_ and obtained 100% alcohol.

## 2. Materials and Methods

### 2.1. Sample Preparation

#### 2.1.1. Preparation of Flower-like SnS_2_

SnCl_4_·5H_2_O (0.525 g) was dissolved into ethylene glycol (80 mL), and then CH_4_N_2_S (thiourea, 0.609 g) was added. The above solution was stirred until dissolved, and dispersed uniformly by ultrasonic wave. Then the mixture was transferred into 100 mL of autoclave and the temperature was kept at 180 °C for 18 h. Finally, the product was washed with ethanol and distilled water.

#### 2.1.2. Preparation of SnS_2_/Bi_2_WO_6_

Bi(NO_3_)_3_·5H_2_O (0.485 g) and Na_2_WO_4_·2H_2_O (0.165 g) were dissolved in 10 mL of ethylene glycol solution, respectively. After dissolving, the two solutions were mixed and dispersed by ultrasonic treatment. SnS_2_ was dissolved in ethylene glycol (60 mL) solution and sonicated for 30 min, then slowly added dropwise to the above solution. The obtained yellow mixture was transferred into a 100 mL of autoclave, and the temperature was kept at 160 °C for 24 h. The product was washed three times with ethanol and distilled water, respectively. The mass ratio of SnS_2_: Bi_2_WO_6_ was 0.025:1, 0.05:1, 0.1:1, and 0.15:1, thus the composites were named as SnS_2_/Bi_2_WO_6_-2.5, SnS_2_/Bi_2_WO_6_-5, SnS_2_/Bi_2_WO_6_-10, and SnS_2_/Bi_2_WO_6_-15, respectively.

### 2.2. Characterization

X-ray diffraction (XRD) patterns of prepared samples were determined on a D/max-3 X-ray diffractometer (Bruker, Germany) using Cu Kα radiation (λ = 0.154056 Å). The detailed surface morphology of catalysts was characterized by employing a Nova Nano SEM450 (FEI, Hillsboro, OR, USA) field-emission scanning electron microscope (SEM) and transmission electron microscopy (TEM) images were obtained with JEOL JEM-2100F (Hitachi, Japan) operated at 200 kV. X-ray photoelectron spectroscopy (XPS) analysis was carried out by a Kratos Axis Ultra DLD spectrometer (VERTEX 70, Bruker, Germany) with a monochromatic Al Kα X-ray source. The property about light absorption of catalysts were determined with a UV-vis spectrophotometer (Hitachi U-3900H, Tokyo, Japan). Photoluminescence (PL) spectra were obtained using a fluorescence spectrophotometer (Hitachi F-7000, Tokyo, Japan). Transient spectra of the testing samples were recorded with time-resolved fluorescence spectroscopy (Edinburgh, Scotland, FS5).

### 2.3. Photoelectrochemical Measurements

The transient photocurrent response, EIS and Mott–Schottky curves were carried out on the electrochemical workstation (CHI660E, Shanghai) in a standard three-electrode system with the Pt mesh as the counter electrode, the Ag/AgCl (saturated KCl) as the reference electrode, and the sample loaded photoelectrode as the working electrode in 0.1 M Na_2_SO_4_ aqueous solution (electrolyte solution) at room temperature. The distance between the counter electrode and the working electrode is 2 cm. Indium tin oxide (ITO) with a 1 cm × 1 cm area photocatalyst was used as the working electrode. The photocurrent measurement of the photocatalyst is measured by several switching cycles of light irradiated by a 300 W xenon lamp (using a 420 nm cut-off filter).

The photoelectrode in this system is prepared as follows: 10 mg of powder sample was dispersed in 0.2 mL of a mixed solution of ethanol and H_2_O (H_2_O:ethanol = 1:1 *v*/*v*), then 5 μL of Nafion solution was added, and the mixture was sonicated for several minutes. Finally, the obtained slurry was coated on an ITO glass substrate in an area of 1 × 1 cm.

### 2.4. Photocatalytic CO_2_ Reduction

Light-driven reduction of CO_2_ was performed in a closed 200 mL quartz glass reactor containing 50 mL of ultrapure water and 0.05 g of photocatalyst at atmospheric pressure. A 300 W Xe arc lamp (PLS-SXE300, Beijing, China) with a 420 nm cut-off filter was positioned 5 cm above the reactor as a visible light source. Before irradiation, high purity CO_2_ gas (99.995%) was bubbled into above suspension for 30 min to expel air and dissolved oxygen. During the whole catalytic process, CO_2_ gas was bubbled into the solution at a rate of 50 mL per minute. Since the efficiency in photocatalytic reduction of CO_2_ is dependent on both the solubility in water and reaction temperature, 4 °C is optimal for this reaction by considering the trade-off between solubility and temperature. The yields of obtained alcohols were determined by sampling the suspension (1 mL) every hour and filtering it with a specific membrane to remove the solid catalysts. The aimed products such as methanol and ethanol were quantitatively monitored with Agilent Technologies 7890A gas chromatography (Shanghai China, FID detector, DB-WAX column). The detailed diagram of the catalytic reaction device is shown in Figure S1.

## 3. Results

### 3.1. Characterization of Materials

**Figure 1:**
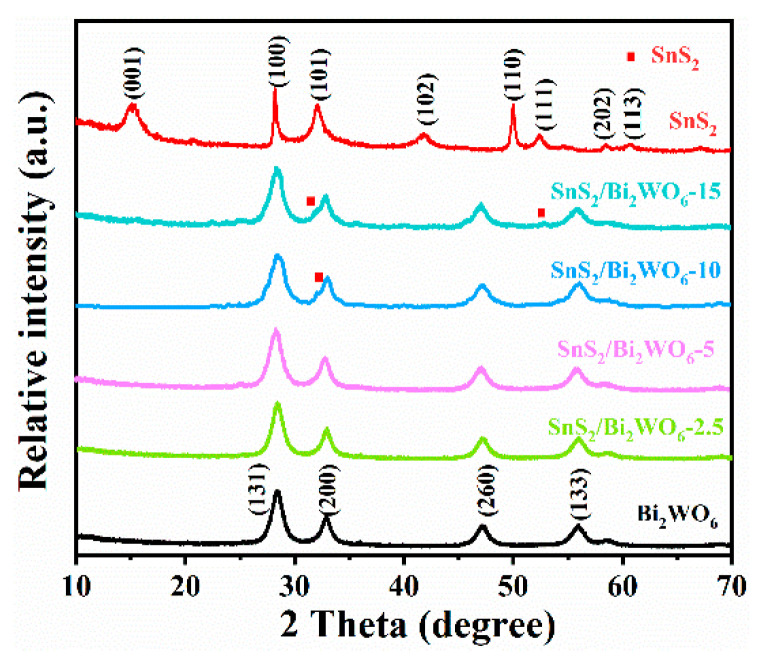
XRD patterns of prepared materials.

Figure 1 shows the XRD patterns of pure SnS_2_, Bi_2_WO_6_ and the composite materials with different mass ratios. The pattern of unmodified Bi_2_WO_6_ suggests all the diffraction peaks are indexed to the orthorhombic phase (JCPDS No. 39-0256). The main diffraction peaks at 28.3°, 32.8°, 47.0°, and 55.9° correspond to the crystal planes of (131), (200), (260), and (133), respectively. Bi_2_WO_6_ has a larger half-width value, and it can be inferred that Bi_2_WO_6_ has a small grain size, which can provide more reactive sites. The pure phase of SnS_2_ mainly has six diffraction peaks, which are consistent with the (001), (100), (101), (102), (110), and (111) planes of the standard card (No. 23-0677). With the increase of the molar ratio of SnS_2_, the diffraction peaks of (101) and (111) crystal planes of SnS_2_ gradually appeared. Since the diffraction peak of SnS_2_ in the composite material is not obvious, the amount of SnS_2_ was increased to 50%, and the obtained XRD patterns are shown in Figure S2. The diffraction peaks of the material with 50% content of SnS_2_ become relatively obvious, especially the diffraction peaks around 15° and 50°. This is due to the different degree of crystallization of materials SnS_2_ and Bi_2_WO_6_, which leads to the obvious difference in the intensity of their diffraction peaks. When the two are combined, the diffraction peaks belonging to SnS_2_ will be easily masked by Bi_2_WO_6_, especially the peaks near the strong diffraction peaks at 28.3° and 32.6°. Therefore, this leads to masking of the two strongest diffraction peaks near these positions in SnS_2_. The half-peak width of (131) composite is slightly larger than that of pure Bi_2_WO_6_ (Figure 1). This phenomenon shows that during the hydrothermal process, due to the interaction between the two materials, the single material will be less likely to aggregate, resulting in a slight decrease in the crystallinity of the material [20]. The BET test in Figure S3 shows that the specific surface areas of Bi_2_WO_6_ and SnS_2_/Bi_2_WO_6_-10 are 25.70 and 37.13 m^2^/g, respectively, which indicates that the specific surface area of the composite is significantly increased, while the pore size is slightly reduced.

**Figure 2:**
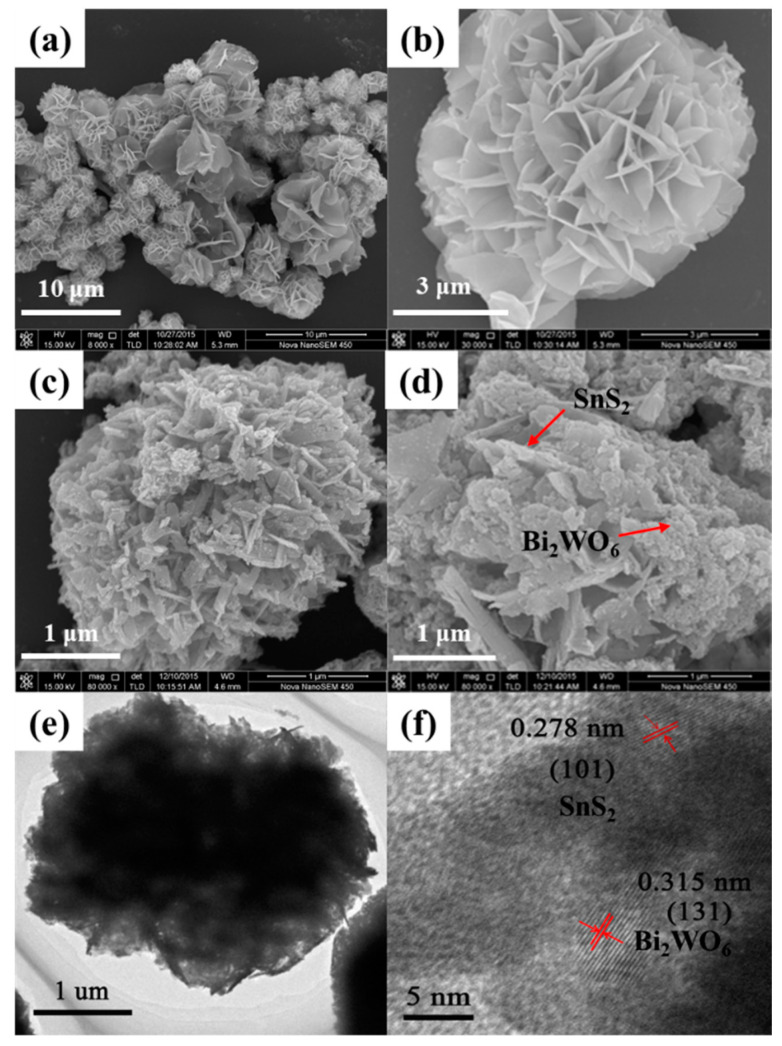
(**a**,**b**) SEM images of flower-like SnS_2_, (**c**,**d**) SEM images of SnS_2_/Bi_2_WO_6_-10, (**e**,**f**) TEM images of SnS_2_/Bi_2_WO_6_-10, red arrows and red lines indicate the lattice spacings.

The flower-like SnS_2_ spheres were prepared by hydrothermal method, as shown in Figure 2a, the diameter distribution ranges from 2 μm to 5 μm. According to Figure 2b, it can be seen more clearly that the spherical SnS_2_ is formed by self-assembly with regular two-dimensional nanosheets. Moreover, the reaction process may be due to the adsorption of ethylene glycol on the precursors of SnS_2_, then the intermolecular force as well as crystal growth orientation make the nanosheets self-assemble into the flower-like spheres. The thickness of nanosheets is about 50 nm, besides there are large pores between the nanosheets, which is conducive to the adsorption of CO_2_. This unique porous structure can provide a large number of catalytic active sites and charge transport channels, therefore, the flower-like SnS_2_ has higher photocatalytic activity than that of SnS_2_ synthesized by traditional method. Figure 2c,d shows the SEM images of the composite SnS_2_/Bi_2_WO_6_-10 obtained by the hydrothermal reaction, while the morphology of nanospheres has no obvious change because of the high dispersion of Bi_2_WO_6_. As can be seen from the TEM image (Figure 2e), the SnS_2_ spheres consist of staggered nanosheets. Two different lattice distances of 0.278 and 0.315 nm can be observed in the high-resolution TEM (HRTEM) image (Figure 2f), corresponding to the (101) plane of SnS_2_ and the (131) plane of Bi_2_WO_6_, respectively. Figure S4 also shows the HRTEM patterns of SnS_2_/Bi_2_WO_6_-10. In addition, it can be seen from the EDS spectrum of SnS_2_/Bi_2_WO_6_-10 (Figure S5) that there are Bi, W, O, Sn, and S elements in the sample, which indicates that the synthesis method does not introduce other impurity elements, and the ratio of SnS_2_ to Bi_2_WO_6_ is approximately close to the theoretical value (0.1:1).

**Figure 3:**
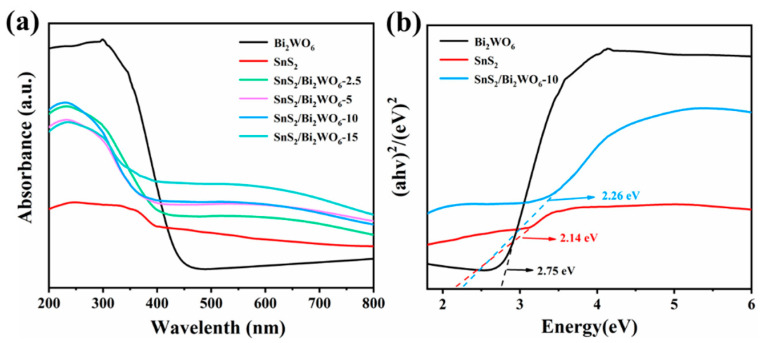
(**a**) Ultraviolet-visible diffuse reflectance spectra, (**b**) corresponding plots of (αhν)^2^ versus energy (hν) for the bandgap energies.

As shown in Figure 3a, an obvious redshift occurs from SnS_2_ to SnS_2_/Bi_2_WO_6_-10, which is beneficial to absorb visible light. The combination of SnS_2_ and Bi_2_WO_6_ makes the bandgap narrow, thus broadening the absorption edges and improving the absorption intensity of visible light. The bandgaps are determined using the Tauc/Davis–Mott model described by the equation: (αhν)^1/n^ = A(hν − E_g_) [21]. The exponent n denotes the natural properties of the material, and the value of n is 0.5 for the direct bandgap. The fitting results (Figure 3b) suggest that the bandgaps of SnS_2_, Bi_2_WO_6_, and SnS_2_/Bi_2_WO_6_-10 are 2.14, 2.75, and 2.26 eV, respectively.

**Figure 4:**
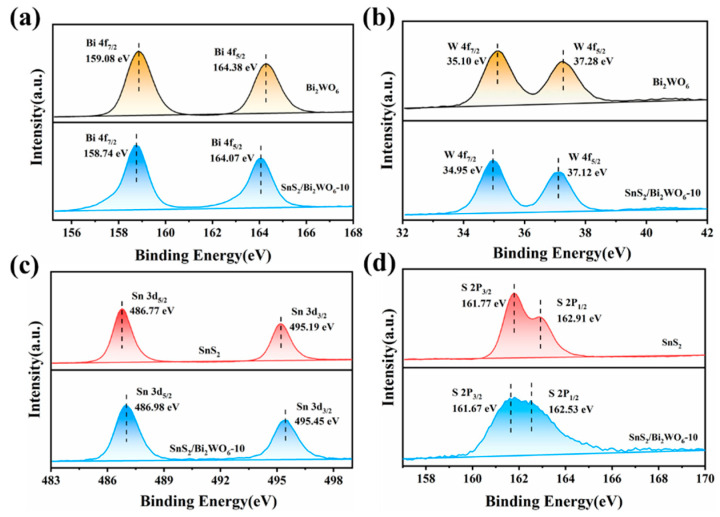
High-resolution XPS spectra of (**a**) Bi 4f, (**b**) W 4f, (**c**) Sn 3d, and (**d**) S 2p.

XPS studies were carried out to investigate the surface elemental compositions and chemical states. As depicted in Figure S6, the XPS survey spectrum of SnS_2_/Bi_2_WO_6_-10 indicates that elements of Bi, W, O, Sn, and S exist in the sample. The Bi 4f spectrum of SnS_2_/Bi_2_WO_6_-10 can be deconvoluted into two peaks with spin orbits of Bi 4f_7/2_ and Bi 4f_5/2_, and the binding energies locate at 158.74 and 164.07 eV assigning to the Bi^3+^ species in Bi_2_WO_6_ lattice [22]. The peaks located at 34.95 and 37.12 eV are assigned to W 4f_7/2_ and W 4f_5/2_ (Figure 4b), respectively, indicating the oxidation state of W^6+^ in SnS_2_/Bi_2_WO_6_-10 composite [23]. Figure 4c displays the high-resolution spectra of Sn 3d, and two characteristic peaks at 486.77 and 495.19 eV ascribe to the inner electrons of Sn 3d_5/2_ and Sn 3d_3/2_ in SnS_2_, demonstrating the feature of Sn^4+^ species in SnS_2_/Bi_2_WO_6_-10 [24]. The peaks located at 161.67 and 162.53 eV are assigned to S 2p_3/2_ and S 2p_1/2_ (Figure 4d), respectively, indicating the oxidation state of S^2−^ in SnS_2_/Bi_2_WO_6_-10 composite. According to the high-resolution XPS spectra, the binding energies of Bi 4f and W 4f move to the direction of lower binding energy in SnS_2_/Bi_2_WO_6_-10, while the peaks of Sn 3d shift to the direction of larger binding energy. The result suggests that the electron density of Bi and W increases, while the electron density of Sn is reduced, which indicates that there is a strong interaction between Bi_2_WO_6_ and SnS_2_, giving rise to the formation of heterojunction.

### 3.2. Improvement of Photogenerated Charge Separation

**Figure 5:**
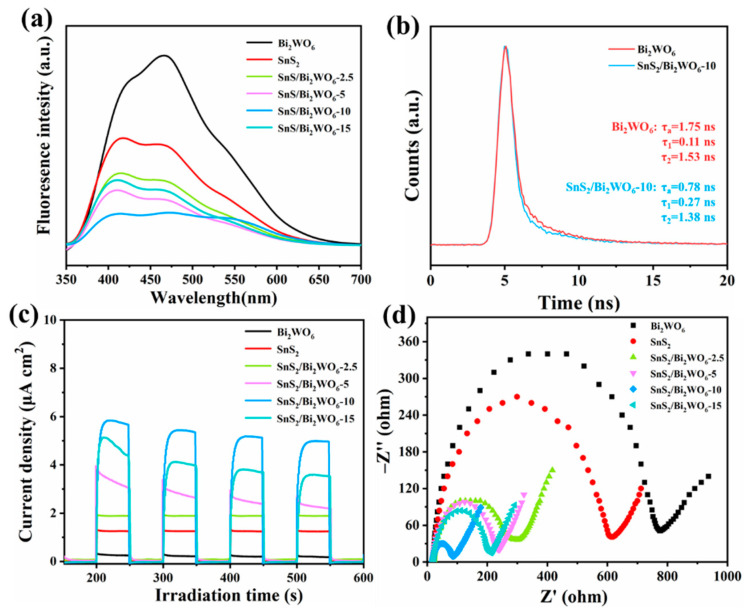
(**a**) Photoluminescence spectra, (**b**) time-resolved PL decay spectra, (**c**) transient photocurrent response, (**d**) EIS Nyquist plots.

Photoluminescence (PL) is an effective as well as simple method to evaluate the probability of photogenerated electron-hole recombination. Figure 5a shows the fluorescence spectra of SnS_2_, Bi_2_WO_6_, and SnS_2_/Bi_2_WO_6_-10 composite catalysts with different mass ratios, SnS_2_ and SnS_2_/Bi_2_WO_6_ with different ratios having similar main peak positions under 300 nm laser excitation. Compared with SnS_2_ and Bi_2_WO_6_ material, the PL intensity of composite materials is significantly reduced due to suppressing the recombination of electron-hole pairs. Therefore, more photoexcited electrons can participate in the reaction of CO_2_ reduction. In addition, the time-resolved PL decay spectra of Bi_2_WO_6_ and SnS_2_/Bi_2_WO_6_-10 are shown in Figure 5b, and the average lifetimes are 1.75 and 0.78 ns, respectively. This indicates that the addition of SnS_2_ will endow the material with effective charge separation, proving the formation of Z-scheme heterojunction between SnS_2_ and Bi_2_WO_6_.

The photocurrent response spectra were tested using a three-electrode system in the electrolyte of 0.5 M Na_2_SO_4_. As can be seen in Figure 5c, in the continuous on-off conversion of visible light, the samples show a relatively stable photocurrent curve, indicating the photogenerated electrons are effectively captured by the photoelectrochemical system. The catalyst with the largest photocurrent is SnS_2_/Bi_2_WO_6_-10, which is about four times that of pure phase SnS_2_. The composite material of SnS_2_/Bi_2_WO_6_ can effectively accelerate the migration of electrons and prolong the lifetime of photogenerated electrons, thus benefitting for improving the efficiency of photocatalytic reduction of CO_2_. Electrochemical impedance spectroscopy (EIS) is presented in Figure 5d, the smaller the radius, the faster the charge transfer on the surface of electrode [25]. From the photocurrent response and EIS spectra, we can see that the Z-scheme heterojunction interface formed by an appropriate amount of SnS_2_ and Bi_2_WO_6_ will facilitate the separation and transport of photogenerated charges. However, when the content of SnS_2_ is further increased, the sheet-like morphology of SnS_2_ will cover Bi_2_WO_6_ nanoparticles, resulting in poor light absorption. In addition, SnS_2_ is prone to recombination of photogenerated electrons and holes due to its narrow bandgap. Therefore, the photocurrent of SnS_2_/Bi_2_WO_6_-15 is lower than that of SnS_2_/Bi_2_WO_6_-10. While the impedance of SnS_2_/Bi_2_WO_6_-15 is higher than that of SnS_2_/Bi_2_WO_6_-10. It can be seen that the radius of SnS_2_/Bi_2_WO_6_-10 in all materials is the smallest, which confirms that SnS_2_/Bi_2_WO_6_-10 possesses an interface that can accelerate the charge transfer, in accordance with the results of PL and photocurrent. At the same time, the Z-scheme heterojunction interface formed with an appropriate amount of SnS_2_ and Bi_2_WO_6_ will facilitate the separation and transport of photogenerated charges. However, when the content of SnS_2_ is further increased, the sheet-like morphology of SnS_2_ will cover Bi_2_WO_6_ nanoparticles, resulting in poor light absorption. In addition, SnS_2_ is prone to recombination of photogenerated electrons and holes due to its narrow bandgap.

### 3.3. Photochemical Reduction of CO_2_

**Figure 6:**
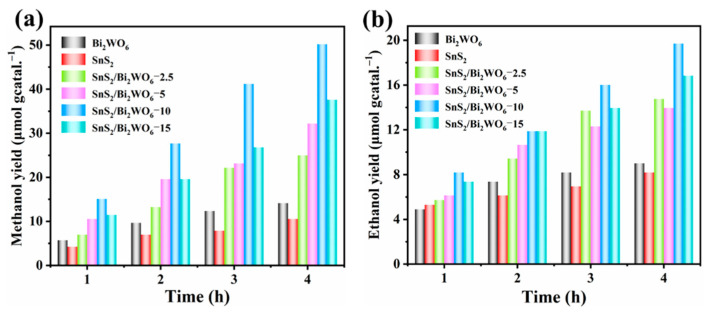
Time dependence of yields of (**a**) methanol and (**b**) ethanol during photoreduction of CO_2_.

Photoreduction of CO_2_ was performed in a closed 200 mL quartz glass reactor containing 50 mL of ultrapure water saturated with CO_2_ and 50 mg of prepared photocatalysts under the irradiation of visible light. After 4 h of photoreaction, only methanol and ethanol were detected as the final products. As presented in Figure 6, with the increase of SnS_2_ in the composites, the yields of methanol and ethanol first increase and then decrease. When the amount of SnS_2_ reaches 10%, the composite photocatalyst shows the highest catalytic activity. The production rate of methanol and ethanol is 50.2 μmol g^−1^ and 19.7 μmol g^−1^ in 4 h respectively, which is nearly 3.3 times that of pure SnS_2_, according to the number of transferred electrons. The yields of methanol and ethanol are shown in Table 1. Moreover, the photocatalytic performance of SnS_2_/Bi_2_WO_6_-10 is superior to that of similar photocatalysts reported in other literature (Table S1). The photocatalytic performance starts to decline with further increase in the amount of SnS_2_, this conclusion is in line with the results of PL, photocurrent, and EIS. When the amount of SnS_2_ reaches a certain level, the catalytic ability drops suddenly, which implies that the excessive SnS_2_ is not conducive to the timely transfer of photogenerated electrons to Bi_2_WO_6_ through Z-scheme heterojunction. In addition, it is worth mentioning that the conduction band (CB) reduction potential of Bi_2_WO_6_ is theoretically insufficient to directly reduce CO_2_ to alcohols. However, after CO_2_ is dissolved into water to form CO_3_^2−^ and other species, the reduction potential is sufficient to reduce carbonate species to alcohols [26].

The control experiments showed that the products of methanol and ethanol were not detected in the dark or without catalyst, indicating that the light source and catalyst are essential factors for photochemical reduction of CO_2_. No carbon-containing product was detected when CO_2_ was replaced by N_2_, suggesting CO_2_ is also a necessary condition for CO_2_ photosplitting. In other words, the element of C in the products is derived from the reactant CO_2_. In order to investigate the stability of SnS_2_/Bi_2_WO_6_-10, the cycling experiment was carried out. At the end of each cycle, the reaction suspension was centrifuged, after washing with distilled water for several times, it was dried in vacuum at 60 °C for next run. As shown in Figure S7, the catalytic performance of SnS_2_/Bi_2_WO_6_-10 shows a slight decrease after four consecutive cycles. Figure S8 is the XRD pattern of SnS_2_/Bi_2_WO_6_-10 after four cycles, which is consistent with fresh one, further proving its good stability.

**Figure 7:**
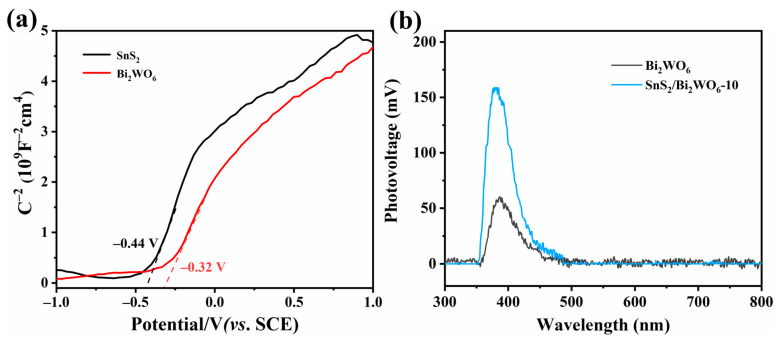
Mott–Schottky plot of (**a**) Bi_2_WO_6_ and SnS_2_, (**b**) surface photovoltage spectra.

Figure 7a shows the Mott–Schottky plots of flower-like SnS_2_ and Bi_2_WO_6_ at voltages from −1.0 to 1.0 V. Classified according to the conductive properties of semiconductors, flower-shaped spherical SnS_2_ and Bi_2_WO_6_ nanoparticle materials belong to n-type semiconductors. The flat band potential (V_fb_) of SnS_2_ is −0.44 V vs. SCE (saturated calomel electrode), which is equivalent to −0.20 V vs. NHE (normal hydrogen electrode), so the CB potential (V_CB_) of flower-shaped spherical SnS_2_ can be calculated to be −0.40 V. The V_fb_ of Bi_2_WO_6_ is −0.32 V vs. SCE, which is equivalent to −0.080 V vs. NHE, so the V_CB_ of Bi_2_WO_6_ can be calculated to be -0.280 V. According to the photocatalytic theory [27], the potentials for photocatalytic reduction of CO_2_ to methanol and ethanol are −0.38 V and −0.33 V (vs. NHE, pH = 7.00), respectively. Therefore, the Z-scheme heterojunction composed of SnS_2_ and Bi_2_WO_6_ has sufficient reduction ability to reduce CO_2_ to methanol and ethanol. In addition, the surface photovoltage test (Figure 7b) further shows that the SnS_2_/Bi_2_WO_6_-10 has higher surface photovoltage after photoexcitation, thus more photogenerated electrons will transfer to the surface active sites, and the photoreduction efficiency of CO_2_ is significantly improved compared to pure phase of SnS_2_ and Bi_2_WO_6_.

**Figure 8:**
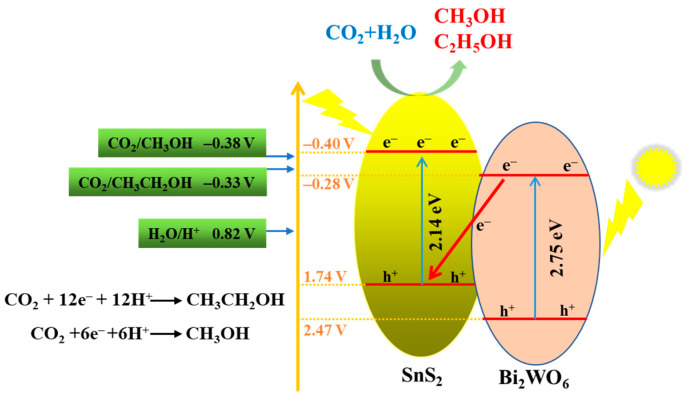
Mechanism diagram of photocatalytic reaction.

Based on the above experimental results and discussion, the possible reaction mechanism is as follows: electrons (e^−^) in the valence bands (VBs) of SnS_2_ and Bi_2_WO_6_ are excited and transfer to the CBs under sunlight irradiation, thus leaving the same number of holes (h^+^) in the VBs of SnS_2_ and Bi_2_WO_6_. Then, the photogenerated electrons on the CB of Bi_2_WO_6_ are transferred to the VB of SnS_2_, subsequently the electrons are excited to the CB of SnS_2_. Therefore, the photogenerated electrons and holes of the SnS_2_/Bi_2_WO_6_ photocatalyst are effectively separated by constructing Z-scheme heterojunction. Based on Equations (1)–(3) [28], the electrons on the CB of SnS_2_ have enough reduction ability to reduce the CO_2_ to methanol and ethanol. This is further demonstrated by Pt photodeposition experiment on SnS_2_/Bi_2_WO_6_-10 (Figure S9). In addition, the Bi_2_WO_6_ sphere is composed of intricate nanosheets, so some intermediates (·CH_3_, ·OCH_3_) may not be converted into methanol immediately, and the dimerization reaction takes place, and finally ethanol can be formed (Figure 8).

CO_2_ + 6H^+^ + 6e^−^ → CH_3_OH + H_2_O − 0.38 V (1)

2CO_2_ + 12H^+^ + 12e^−^ → C_2_H_5_OH + H_2_O − 0.33 V (2)

H_2_O + 4h^+^ → O_2_ + 4H^+^ 0.82 V (3)

## 4. Conclusions

Here, flower-like SnS_2_ microspheres were successfully prepared by hydrothermal method, and SnS_2_/Bi_2_WO_6_ composites with different mass ratios were synthesized. Among them, SnS_2_/Bi_2_WO_6_-10 exhibited the highest catalytic activity in the photochemical reduction of CO_2_ without sacrificial agent, and only methanol and ethanol were detected as reduction products. The improvement of catalytic activity is attributed to the formation of Z-scheme heterojunction between SnS_2_ and Bi_2_WO_6_. Under sunlight irradiation, the photogenerated electrons in the CB of Bi_2_WO_6_ will be transferred to the VB of SnS_2_. Therefore, the photogenerated electrons and holes of the SnS_2_/Bi_2_WO_6_ photocatalyst are effectively separated, which effectively inhibits the recombination of photogenerated electron-hole pairs and expands the light absorption range. Finally, the yield of CO_2_ photoreduction to alcohol products was 3.3 times higher than that of SnS_2_ in pure water.

## Figures and Tables

**Table 1:** 4 h yield of methanol and ethanol.

Sample	Yield of Methanol (μmol g^−1^)	Yield of Ethanol (μmol g^−1^)
Bi_2_WO_6_	14.11	9.00
SnS_2_	10.55	8.18
SnS_2_/Bi_2_WO_6_-2.5	24.97	14.76
SnS_2_/Bi_2_WO_6_-5	32.18	13.94
SnS_2_/Bi_2_WO_6_-10	50.20	19.70
SnS_2_/Bi_2_WO_6_-15	37.58	16.82

## Data Availability

Not applicable.

## Supplementary Materials

The following supporting information can be downloaded at: https://www.mdpi.com/article/10.3390/nano12122030/s1. Figure S1: Reaction device diagram. Figure S2: XRD spectra of SnS_2_/Bi_2_WO_6_-50. Figure S3: (a) N_2_ adsorption-desorption isotherms, and (b) pore size distribution profiles of Bi_2_WO_6_ and SnS_2_/Bi_2_WO_6_-10. Figure S4: HRTEM spectra of SnS_2_/Bi_2_WO_6_-10. Figure S5: EDS spectrum of SnS_2_/Bi_2_WO_6_-10. Figure S6: XPS survey spectra of Bi_2_WO_6_ and SnS_2_/Bi_2_WO_6_-10. Figure S7: Cycling tests with catalyst SnS_2_/Bi_2_WO_6_-10 for 4 h irradiation. Figure S8: XRD patterns before and after recycling. Figure S9: HRTEM spectra of 1%Pt-SnS_2_/Bi_2_WO_6_-10. Table S1: The comparison of CO_2_ photoreduction performance. References [29,30,31,32,33,34,35] are cited in the supplementary materials.
