# Supplementary material for: Z-Scheme Heterojunction of SnS2/Bi2WO6 for Photoreduction of CO2 to 100% Alcohol Products by Promoting the Separation of Photogenerated Charges
Source: Nanomaterials (Basel). 2022 Jun 13;12(12):2030. doi: 10.3390/nano12122030 (PMC9229445; doi:10.3390/nano12122030)
Supplement: Supplementary file 1 [file nanomaterials-12-02030-s001.zip › nanomaterials-1723910-supplementary.pdf]

# Supporting Information

# Z-Scheme Heterojunction of $SnS_2/Bi_2WO_6$ for Photoreduction of $CO_2$ to 100% Alcohol Products by Promoting the Separation of Photogenerated Charges

**Yong Xu, Juanjuan Yu, Jianfei Long, Lingxiao Tu, Weili Dai * and Lixia Yang**

Key Laboratory of Jiangxi Province for Persistent Pollutants Control and Resources Recycle,
School of Environmental and Chemical Engineering, Nanchang Hangkong University, Nanchang 330063, China; xu_yong001@163.com (Y.X.); qddxhhh_1@sina.com (J.Y.); 15007047545@139.com (J.L.); 2002085700109@stu.nchu.edu.cn (L.T.); zgay20080808@126.com (L.Y.)
* Correspondence: wldai81@126.com

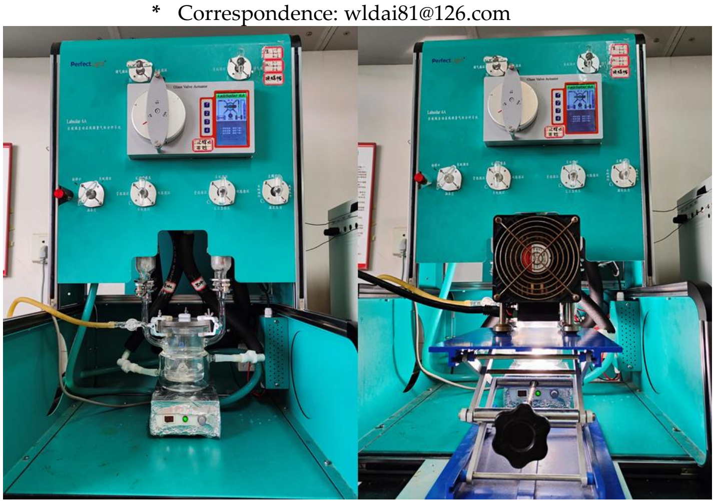

**Figure S1** Reaction device diagram.

The reaction device in this work is shown in Figure S1. In the photocatalytic reaction, high-purity $CO_2$ gas is continuously bubbling into the reactor from the gas inlet. The reactor is connected to condensed water and the temperature is kept at 4 °C.

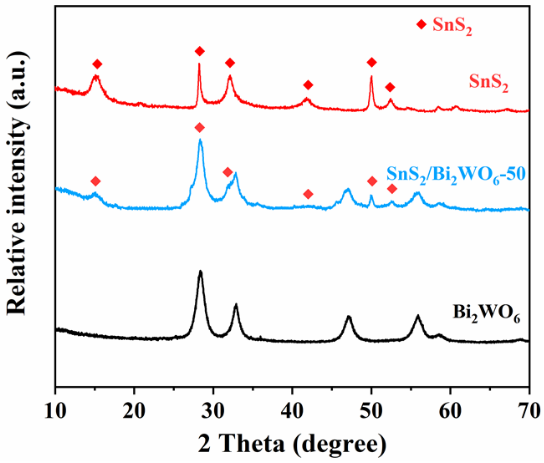


**Figure S2** XRD spectra of $SnS_2/Bi_2WO_6$-50.

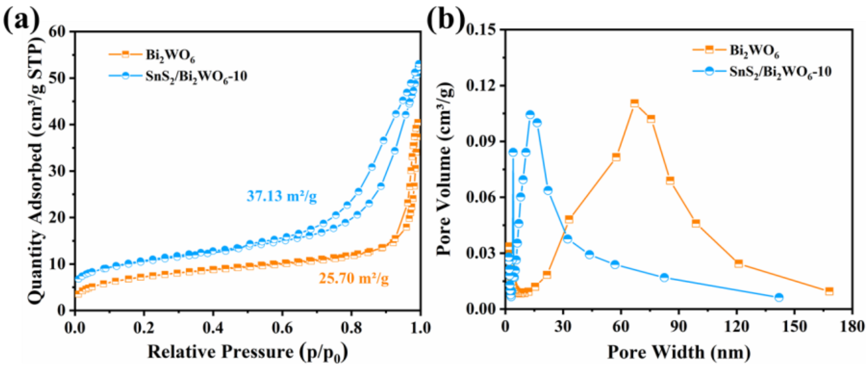


**Figure S3** (a) $N_2$ adsorption-desorption isotherms, and (b) pore size distribution profiles of $Bi_2WO_6$ and $SnS_2/Bi_2WO_6$-10.

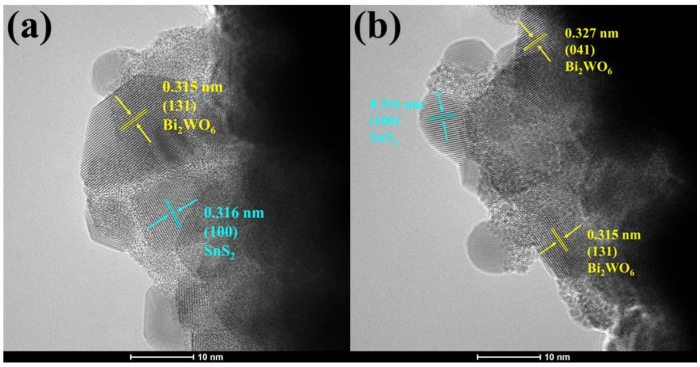


**Figure S4** HRTEM spectra of $SnS_2/Bi_2WO_6$-10.

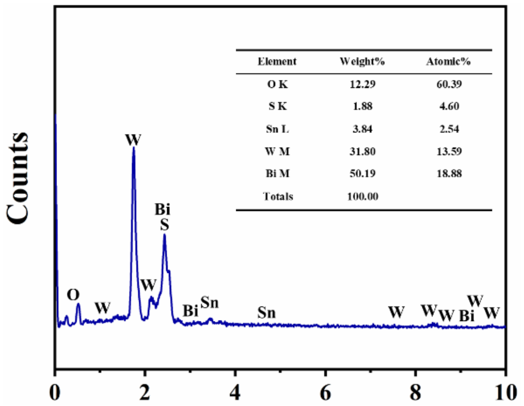


**Figure S5** EDS spectrum of $SnS_2/Bi_2WO_6$-10.

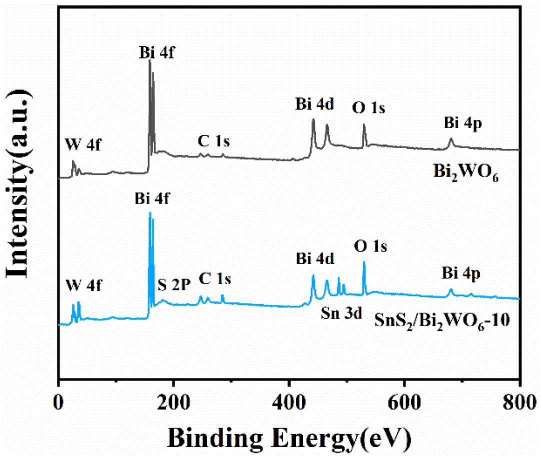


**Figure S6** XPS survey spectra of $Bi_2WO_6$ and $SnS_2/Bi_2WO_6$-10.

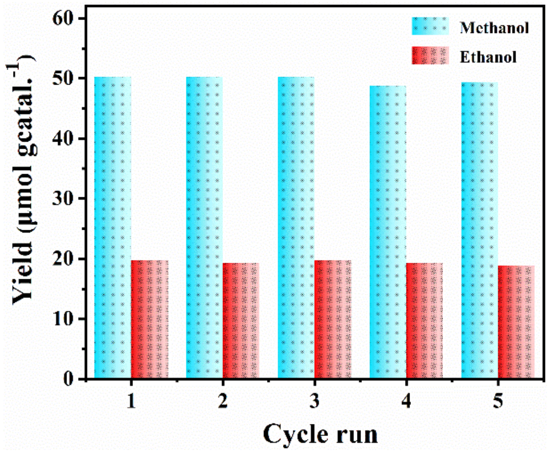


**Figure S7** Cycling tests with catalyst $SnS_2/Bi_2WO_6$-10 for 4 h irradiation.

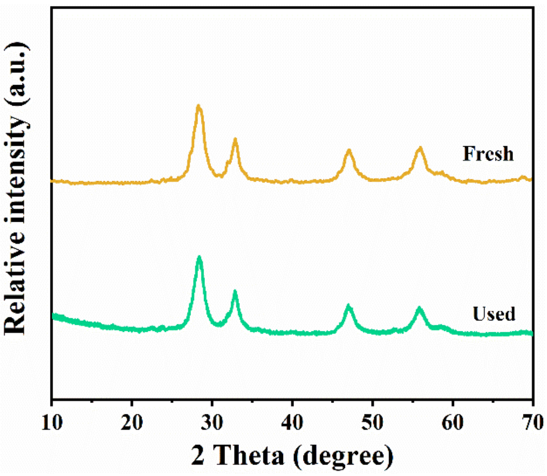


**Figure S8** XRD patterns before and after recycling.

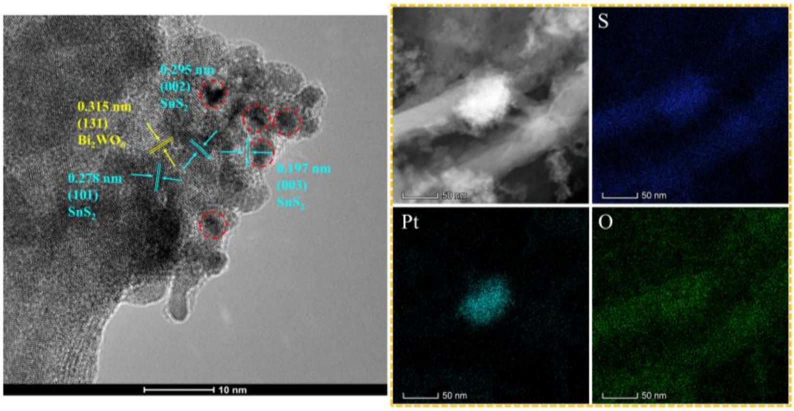


**Figure S9** HRTEM spectra of 1%Pt-$SnS_2/Bi_2WO_6$-10.

**Table S1** The comparison of $CO_2$ photoreduction performance

| Photocatalyst | Light sources | Main products | Formation rate (μmol $g^{-1}$ $h^{-1}$) | Ref. |
|---|---|---|---|---|
| $Bi_2WO_6/SnS_2$ | λ ≥ 420 nm | $CH_3OH$<br>$C_2H_5OH$ | 12.55<br>4.93 | This work |
| $Bi_2MoO_6$ | λ ≥ 420 nm | $CH_3OH$ | 6.2 | 29 |
| 10% $MoS_2/TiO_2$ | 350 W Xe lamp | $CH_4$<br>$CH_3OH$ | 2.86<br>2.55 | 30 |
| $CuIn_2S_4$–0.33/ $TiO_2$ | 300 W Xe lamp | $CH_4$ | 1.14 | 31 |

| | | | | |
|---|---|---|---|---|
| Ag–Mn–N/$TiO_2$ | 300 W Xe arc lamp | $CH_3OH$ | 0.53 | 32 |
| PPy/$Bi_2WO_6$ | 300 W Xe lamp | $CH_3OH$ | 14.1 | 33 |
| Pd/$Gd(OH)_3$ | 500 W high-pressure Xe lamp | $CH_3OH$ | 0.74 | 34 |
| $CeO_2$-$TiO_2$ | 500 W Xe lamp | $CH_3OH$ | 6.3 | 35 |
